# Resolving Structure and Mechanical Properties at the Nanoscale of Viruses with Frequency Modulation Atomic Force Microscopy

**DOI:** 10.1371/journal.pone.0030204

**Published:** 2012-01-25

**Authors:** David Martinez-Martin, Carolina Carrasco, Mercedes Hernando-Perez, Pedro J. de Pablo, Julio Gomez-Herrero, Rebeca Perez, Mauricio G. Mateu, Jose L. Carrascosa, Daniel Kiracofe, John Melcher, Arvind Raman

**Affiliations:** 1 Departamento Física de la Materia Condensada, Universidad Autónoma de Madrid, Madrid, Spain; 2 Centro Nacional de Biotecnología, CSIC, Madrid, Spain; 3 Centro de Biología Molecular “Severo Ochoa” (CSIC), Madrid, Spain; 4 School of Mechanical Engineering and Birck Nanotechnology Center, Purdue University, West Lafayette, Indiana, United States of America; Massachusetts Institute of Technology, United States of America

## Abstract

Structural Biology (SB) techniques are particularly successful in solving virus structures. Taking advantage of the symmetries, a heavy averaging on the data of a large number of specimens, results in an accurate determination of the structure of the sample. However, these techniques do not provide true single molecule information of viruses in physiological conditions. To answer many fundamental questions about the quickly expanding physical virology it is important to develop techniques with the capability to reach nanometer scale resolution on both structure and physical properties of individual molecules in physiological conditions. Atomic force microscopy (AFM) fulfills these requirements providing images of individual virus particles under physiological conditions, along with the characterization of a variety of properties including local adhesion and elasticity. Using conventional AFM modes is easy to obtain molecular resolved images on flat samples, such as the purple membrane, or large viruses as the Giant Mimivirus. On the contrary, small virus particles (25–50 nm) cannot be easily imaged. In this work we present Frequency Modulation atomic force microscopy (FM-AFM) working in physiological conditions as an accurate and powerful technique to study virus particles. Our interpretation of the so called “dissipation channel” in terms of mechanical properties allows us to provide maps where the local stiffness of the virus particles are resolved with nanometer resolution. FM-AFM can be considered as a non invasive technique since, as we demonstrate in our experiments, we are able to sense forces down to 20 pN. The methodology reported here is of general interest since it can be applied to a large number of biological samples. In particular, the importance of mechanical interactions is a hot topic in different aspects of biotechnology ranging from protein folding to stem cells differentiation where conventional AFM modes are already being used.

## Introduction

Millions of years of evolution have converted viruses into nanomachines optimized to carry out complex functions with a minimalistic structure [1]. Viruses are masterpieces of nanoengineering with a basic common architecture that consists of the capsid – a protein shell made up of repeating protein subunits- which packs within it the viral genome. Beyond this basic architecture, viruses can have further elaborations such as protein collars, tails, connectors, lipid coats, surface receptors, enzymes, and molecular motors. With this simple structure, viruses are able to withstand tremendous internal pressure [2], osmotic shocks [3], [4] and chemical agents while maintaining their viability. To the materials engineer or nanotechnologist, viruses are perfectly defined organic nanoparticles which are commonly used as scaffolds or nano-vectors [5]. Because their structure can be directed by tailored evolution and their production can be commercially viable, viruses are becoming the basis of whole new approaches to the manufacture of nanomaterials far beyond applications in biology and medicine. There has been a significant increase in the use of viruses for the template assembly of nanomaterials with applications from vaccines to electronics [6], [7], [8]. The extreme resistance of virus particles to the external media conditions has triggered the attention over the mechanical properties of viruses [9] and their relationship with their structure [10], [11]. These studies have used *in vitro* AFM as the main tool to characterize the topography and the stiffness of the virus particles, a current hot topic in physical virology [12]. While AFM characterization of mechanical properties is usually carried out by nanoindentation [13] in this work we present a new method that allows obtaining, in addition to the topography, spatially resolved maps of flexibility.

In *vitro* AFM is a powerful technique that provides images of biological specimens with a resolution far beyond optical microscopy. AFM can achieve molecular or even atomic resolution [14] in the classical contact mode when applied to hard samples where the friction tip-sample force is not very relevant. The so called purple membrane is a good example of a bio-sample that can be scanned with molecular resolution using contact mode [15]. On the contrary, small virus particles (25–50 nm), which are particular but very relevant type of weakly attached (to a substrate) samples, cannot be scanned in contact mode. In fact, amplitude modulation (AM-AFM) (also known as Tapping Mode™) imaging of small particles turns out to be a very demanding task that in many cases requires special types of cantilever [16]. AM-AFM is the most widespread dynamic AFM mode that is present in almost every commercial AFM microscope [17]. Jumping mode, [18] an AFM technique that is midway between contact and AM-AFM, is less restrictive, but again the cantilever stiffness has to be low, otherwise the residual lateral forces [19] can still move the virus particles. Larger size viruses, as is the case of the Giant Mimivirus [20], can be scanned using AM-AFM with good resolution. Nevertheless, the lateral forces in a 750 nm virus particle (well above the tip size) are not very relevant and scanning this type of sample is similar to scanning the above described membranes. The restrictions observed in both jumping mode and AM-AFM methods, as we shall see, can be overcome using FM-AFM.

**Figure 1:**
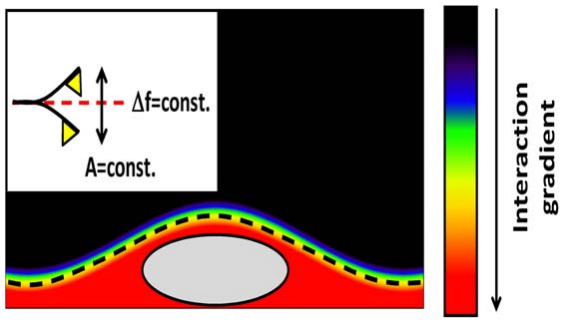
*In liquid* FM-AFM basis. Artistic draw of a virus particle immersed in a buffer solution and adsorbed on a surface. The colored environment represents the total gradient of short and long-range interactions between the cantilever tip and the sample. FM-AFM follows the constant gradient lines to get the sample topography.

Figure 1 outlines the basic features of FM-AFM. A cantilever is oscillated at its resonance frequency, which changes as the tip approaches the surface due to the tip-sample interaction. This magnitude, usually referred to as the frequency shift, is the main control parameter in FM-AFM. For small oscillation amplitudes (linear regime), the frequency shift is proportional to the force gradient (stiffness) of the tip-sample interaction. The topography of the sample is obtained by adjusting the tip sample distance (by means of the z piezoelectric actuator) to track a certain resonance frequency. Therefore a topography image in FM-AFM, in the linear regime, is a map of constant force gradient (dashed line in fig. 1). More generally, for larger oscillation amplitudes (nonlinear regime), the FM-AFM topography image is a more complex mapping of the *conservative* interaction potential [21]. An additional feedback loop is used to keep the cantilever oscillation amplitude constant at the drive frequency by adjusting the drive amplitude. The record of the drive amplitude over the course of a scan provides a second image where variations in the drive amplitude are proportional to the energy dissipated by *nonconservative* tip-sample interactions (viscoelasticity, friction, hysteretic tip-sample forces). The drive signal image is known as a compositional map, because, unlike the frequency shift, the drive signal is free to vary during the course of a scan according to the composition of the sample. Please observe that the macroscopic radius of the tip is not directly related with the final resolution of the probe but with the decay length of the interaction. In AFM there are both long and short range interactions, both of them can be used to obtain the topography but only short ones provide atomic resolution. FM-AFM provides a way to access these short range interactions.

## Results

**Figure 2:**
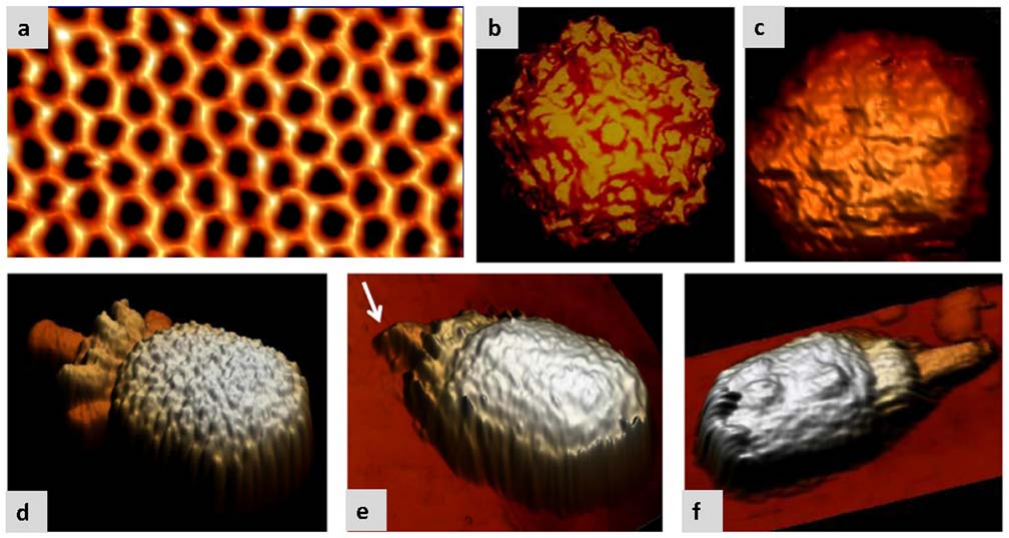
High resolution FM-AFM. Seeing individual features. (a) Image of a muscovite mica surface (001) showing true atomic resolution. The mica was immersed into a physiological buffer to ensure that under these conditions we are able to obtain true atomically resolved images. Parameters of the image: cantilever oscillation amplitude 0.5 nm and frequency shift 36 Hz, scan speed 800 nm/s, Resonance frequency 130 KHz, nominal spring constant 40 N/m. (b) View of a MVM particle oriented with a 5-fold symmetry axis on top as obtained from a molecular surface model derived from crystallographic data [36] as rendered in [11]. The capsid diameter is 25 nm. (c) FM-AFM topographic image of an individual MVM particle in the same orientation shown in (b).Virus particles were adsorbed on a glass surface immersed into a physiological buffer (PBS, 137 mM NaCl, 2.7 mM KCl, 1.5 mM NaH_2_PO_4_, 8.1 mM KH2PO_4_). Parameters of the image: cantilever oscillation amplitude 0.9 nm and frequency shift 30 Hz, speed 350 nm/s, Resonance frequency 19 KHz. Calibrated spring constant of 0.6 N/m. (d) Cryo-EM model of the φ29 bacteriophage [24] (equatorial diameter ∼45 nm) after a *dilation* process using a size tip of 0.2 nm. Reconstruction techniques are extremely precise but require the merging and averaging of a large set of images of individual viruses and, consequently, they tend to neglect individual particle features. (e) FM-AFM topographic image of an individual φ29 particle in TMS buffer (50 mM Tris pH7.8, 10 mM Mg Cl_2_, 100 mM Na Cl). This virus presents a damaged tail (see white arrow). Parameters of the image: cantilever oscillation amplitude 1.0 nm and frequency shift 31 Hz, scan speed 350 nm/s, Resonance frequency 35 KHz, calibrated spring constant of 1.8 N/m. (f) Another φ29 virus particle with a clear and well resolved tail but with some material bound to its head, forming extra protrusions. Parameters of the image: cantilever oscillation amplitude 1.0 nm and frequency shift 25 Hz, scan speed 350 nm/s, Resonance frequency 19 KHz calibrated spring constant of 0.6 N/m.

Figure 2a shows an atomically resolved image of a mica surface in a buffer solution [22] and serves as a benchmark of the performance of our system when working in FM-AFM. We take this result as the upper bound for the resolution of FM-AFM that is not reached by the other AFM working modes. Figure 2b is a view of the parvovirus minute virus of mice (MVM), oriented with a 5-fold symmetry axis on top as obtained from a molecular surface model derived from X-ray data [23]. According to the data provided by this technique, the parvovirus capsid can be inscribed into a 25 nm diameter sphere and it is formed by 60 structurally equivalent subunits arranged in a simple (T = 1) icosahedral symmetry. Figure 2c is a FM-AFM topographic image of a MVM particle adsorbed on a functionalized glass surface. Most of the structural features in fig. 2b (including the pentameric arrangement of the structural capsomeric units) can be readily observed in fig. 2c where features as small as 1 nm can be discerned in the image. Figure 2d shows a three-dimensional volume reconstruction of the φ29 viral particle derived from a large set of images taken by Cryo-EM [24]. It has a prolate head around 55 nm long, with an equatorial diameter of about 45 nm, which presents a tail structure in a singular vertex. It is important to note that in this case, as in the case of X-ray data, the features observed in the specimen are the result of a reconstruction process that hide singular features of individual viruses, as these methods are based on heavy averaging to increase the periodic common features of these specimens. In order to make a more objective estimation of the degree of similarity with AFM images, the cryo-TEM derived volume was subjected to a dilation using a tip of 0.2 nm [25] that takes into account the finite dimensions of the tip apex. Figures 2e and 2f are FM-AFM images of two different φ29 virus. In these images, the three main domains of the virus particle are clearly defined: the prolate head, the collar region and the tail. Besides, fine structural features, as the corrugation of the capsid due to the capsomeric subunit arrangement and, the periodic aspect of the collar due to the presence of the collar appendages can be also clearly seen. Furthermore, a number of individual details are captured in the images, for instance the virus in fig. 2e has the tail bent, probably due to distortions derived from the adsorption process, while the fig. 2f shows the presence of two particular protrusions on the surface of the capsid. As they are located at the interface of the equatorial region with the icosahedral caps, it is tempting to suggest that they might be derived from some kind of aggregate of the virus fibers, but we cannot exclude that they could be caused by small particles floating in the buffer solution that were finally adsorbed onto the virus surface. Also, it is important to note that in the distal part of the cylindrical tail extension there is a cone-shaped knob that has been previously described in some cryo-EM reconstructions of φ29 viral particles prepared under specific conditions, that might be related to the release of the DNA from the capsid [26].

**Figure 3:**
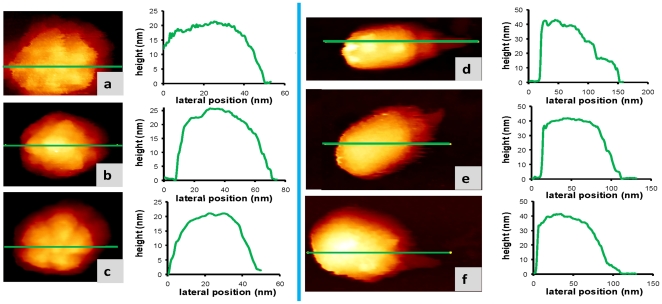
Quantifying virus particle height: (a,c) are MVM virus particles showing the 5-fold symmetry. (b) is a MVM virus particle adsorbed along the 3-fold symmetry. Fig d-f are φ29 bacteriophages. For each topography image we have included a profile that informs about the height of the virus particle that coincides with the expected height as obtained by SB techniques. For the sake of comparison we are showing the images with a simple top-view rendering with a pseudocolor table that encodes the particle heights. Figures 3a, 3d and 3e are the same as figs. 2c, 2f and 2e respectively. The calibrated cantilever stiffness for images 3a and d is 0.6 N/m, oscillation amplitude 1 nm, frequency shift about 25 Hz. The calibrated cantilevers stiffness for rest of the images was 1.8 N/m, the oscillation amplitude 1 nm and the frequency shift was about 30 Hz. All the images were acquired with 350 nm/s scanning speed.

Image processing is an important feature for EM and crystallography. While this is also the case for AFM the image processing required is less intensive. In fig. 2 we have chosen a projection rendering of the virus images because an adequate lightning tends to enhance the virus edges. Figure 3 a,d,e depicts the virus images shown in fig. 2 c,e,f but just with a pseudocolor table that represents the topographical height. In addition we have added 2 new images for the MVM (fig. 3b and c) and one more for the φ29 (fig. 3f). In order to quantify the virus height for each topography image we show the corresponding line profile.

An outstanding feature of FM-AFM is its capability to image soft samples with a variety of cantilevers. We obtained similar images to these shown in fig. 2 and 3 with cantilever stiffness ranging from 0.6 N/m up to 40 N/m (see image supporting information fig. S1). On the contrary, acquiring AFM images with Jumping mode or AM-AFM requires cantilevers with very low stiffness. This feature can be very relevant for many applications. For instance, in order to achieve the best sensitivity when measuring mechanical properties, a cantilever stiffness as similar as possible to the sample stiffness should be used.

## Discussion

A key feature of FM-AFM in vacuum and ambient environments is that the response to conservative and non-conservative (dissipative) tip-sample interactions are decoupled in the frequency shift and the drive signal, respectively. Consequently the topography image is a reflection of purely conservative interactions while the drive signal measures the nonconservative. For this reason, the drive amplitude channel is usually referred to as the *“dissipation”* channel in FM-AFM. As we shall see, this decoupling is not generally the case in liquids and *“drive amplitude”* and *“tip-sample dissipation”* are not synonymous.

**Figure 4:**
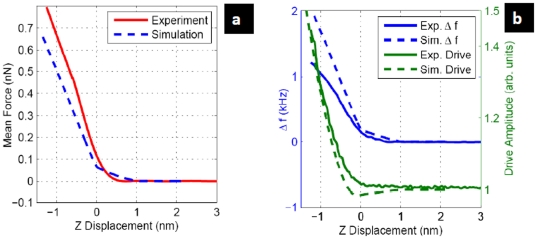
*In liquid* FM-AFM basis. (a) Experimental (solid line) and simulated (dashed line) average deflection (mean force) of the cantilever as the tip approaches the sample surface. The horizontal axis is the piezo elongation. (b) Experimental (solid lines) and simulated (dashed lines) frequency shift and drive amplitude as the tip approaches de sample surface. The horizontal axis is the piezo elongation. All the magnitudes in (a) and (b) were acquired simultaneously. Oscillation amplitude 1.2 nm., calibrated spring constant of 0.6 N/m.

The continuous line in fig. 4a is the experimental cantilever average deflection as a function of the tip sample distance (the magnitude that is accessible in the experiments is the scanner elongation along the z direction, that is the variation in the tip sample distance (absolute positions always require a theoretical model). At large z distance the average force is zero and as the tip approaches the sample it first growths with a smooth elbow and then linearly. This experimental curve can be nicely reproduced by assuming the presence of solvation layers [27], at forces of about 20 pN, that introduce a weak interaction before the mechanical contact between the tip and the surface. Importantly, the hard tip-surface contact occurs at forces below 100 pN, right at the end of the elbow region where we have set the origin for the z scale. The optimum image conditions are found at these forces, all the images in the manuscript are taken in the range of 40–80 pN. The dashed line shows a simulated curve in which a simple conservative model for both the solvation layer and sample forces is incorporated (see Text S1 for full details).

In fig. 4b we are representing the frequency shift (blue line, left scale) and the cantilever driving amplitude (green line, right scale). The average cantilever deflection, the frequency shift and the cantilever driving amplitude has been simultaneously acquired in a single z excursion. As in fig. 4b continuous lines are for the experimental results and dashed lines are the result of simulations. The central quantity in FM-AFM experiments is the frequency shift, as it is used to fix the tip-sample separation and generate the topography image. In the virus images reproduced in figs. 2 and 3, the frequency shift is set at about 40 Hz. Imaging at a higher frequency shift can cause the virus particles to deform under the force from the tip or even become displaced or damaged. Also, although it is still possible to acquire images at a smaller frequency shift, where applied forces are extremely weak, the resolution is degraded because the tip is essentially probing the solvation layers at this point. To achieve optimum resolution the tip must penetrate these layers and start to graze the surface atoms of the sample. Near the grazing contact with the sample, the amplitude of the oscillation will be large compared to indentation of the sample and the probe will be operating in a nonlinear regime.

**Figure 5:**
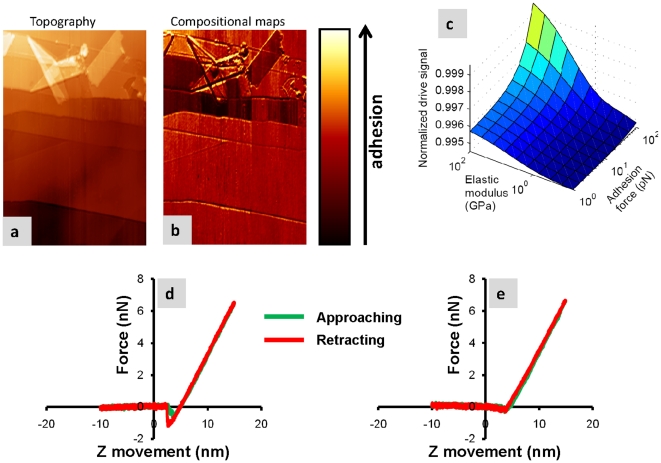
Adhesion force as a source of dissipation. (a) Bare HOPG topography taken in FM-AFM in the same buffer solution used for the MVM viruses. (b) Dissipation map simultaneously taken with a. (d) and (e) are 2 force distance curves taken in the bright and dark regions of the dissipation respectively. As can be seen from the curves there is a clear correspondence between adhesion and dissipation. Cantilever oscillation amplitude 1.0 nm and frequency shift 35 Hz, scan speed 350 nm/s, Resonance frequency 21 KHz, calibrated spring constant of 0.6 N/m. (c) Simulation of the drive signal versus elastic modulus and adhesion. For a given sample with a homogeneous elastic modulus, the drive signal increases as the adhesion gets higher.

In order to understand the contrast within drive amplitude images (also known as dissipation images in the literature) for a surface immersed in liquid, we start by scanning a bare graphite sample. Figures 5a and b show the topography and drive amplitude images obtained in this sample immersed in the same buffer solution used for imaging the MVM virus. The drive amplitude image shows regions with different contrast. In traditional FM-AFM, this contrast in drive amplitude would indicate that the different sample regions had differing amounts of tip-sample dissipation (non-conservative tip-sample interactions). In the present case we do not expect that there should be any difference in dissipation between the regions. However, by acquiring force vs. distance static curves (figs. 5d and e) it can be easily shown that these regions exhibit very different conservative adhesion force (probably due to different van der Waals forces arising from the adsorption of molecules from the buffer solution) but the stiffness, given by the slope of the curve near the contact region, is the same. In particular, the darkest regions show almost zero adhesion force. Therefore, we conclude in this case that the drive amplitude is correlated to the conservative van der Waals forces. Figure 5c shows a computer simulated surface map of the drive signal as a function of the sample stiffness and the adhesion force that confirm the variation of the drive signal for regions with the same stiffness but different adhesion force. Before discussing the reason for this curious result, we provide a second example.

**Figure 6:**
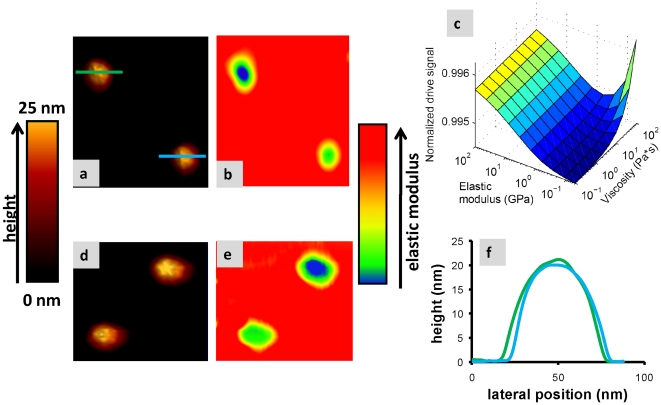
Stiffness as a source for dissipation. (a) and (d) FM-AFM topographies showing, each of them, two virus MVM particles containing genetic material. In both images the particles have different spring constant since they are sitting on different fold-symmetries [10], [11]. (b) and (e) Drive amplitude maps taken simultaneously with a and d. Since the adhesion is negligible, the compositional map is only plotting the elastic modulus. As expected for the different fold-symmetries, the virus showing the 5 fold-symmetry has lower stiffness than the other one which is showing the 3 fold-symmetry. Furthermore the viruses are softer than the substrate. (c) Simulation of the drive signal versus elastic modulus and viscosity. If the adhesion is close to zero, the drive signal is basically the elastic modulus of the sample, with the exception of samples that could have an extremely high viscosity orders of magnitude higher that the expected for virus particles. Fig c despites the corresponding profiles across the two virus particles observed in fig. 6a. Cantilever oscillation amplitude 1.0 nm and frequency shift 32 Hz, scan speed 350 nm/s, Resonance frequency 21 KHz, calibrated spring constant of 0.6 N/m.

Figure 6a and d displays FM-AFM topographies, each of them with two MVM virus particles, adsorbed on a graphite substrate with negligible adhesion along the 5 fold symmetry (upper particle) and 3 fold symmetry (lower particle). It is know that full MVM virus particles (containing genetic material) present anisotropic stiffness. By performing individual indentation curves on the particles it has been found stiffness of 0.6 N/m and 0.8 N/m for the 5 and 3 fold symmetry respectively [10], [11]. Figures 6b and e are the corresponding drive amplitudes maps simultaneously acquired with the topography images. There is an obvious striking correspondence between the variations of this magnitude and what it is expected for the virus stiffness. Figure 6f despites the corresponding profiles across the two virus particles observed in fig. 6a. As can be seen the topographical height of both viruses is basically the same indicating the absence of cross talking between the topography and the compositional map images. This is also the case for fig. 6d. In order understand this correspondence we have simulated the variation of the driving signal as a function of the sample stiffness (elastic modulus) and its viscosity (assuming a sample with low adhesion). The drive signal is actually invariant with respect to viscosity except in the combination of extremely low elasticity and high viscosity. For moderate to low viscosities, as expected for virus particles, the driving signal is monotonically increasing with respect to the elastic modulus.

The implication is that drive amplitude images in liquids are not related to tip-sample dissipation, as in air or vacuum conditions, but maps of the short range conservative mechanical properties (local elasticity, conservative van der Waals forces, conservative electrostatic forces, etc.) of the surface with resolution at the nanometer scale.

One reason for the difference in the FM-AFM drive signal images in liquid compared to vacuum environments can be traced to recent findings in AM-AFM in liquids [28], [29]. In the absence of tip-sample interactions, the probe oscillates with a pure tone at the drive frequency. However, when the probe encounters nonlinear interactions from the sample, the oscillation waveform is populated with higher frequency content (higher harmonic distortions and even contributions from higher vibrational eigenmodes of the probe). The energy that is supplied to the probe from the drive is partially dissipated into the liquid medium by the higher frequency components of the oscillation, even when the cantilever is driven at small oscillation amplitude (<1 nm). Although this higher frequency content is always present in dynamic AFM to some extent, it is only when operating in liquids that the energy dissipated by higher frequency oscillations can be much larger than the energy dissipated by nonconservative tip-sample interactions [29]. This is because the lower quality factors in liquid enhance the energy transfer between eigenmodes. Unlike the tip-sample dissipation, the energy dissipated in the higher frequency oscillations can be result from purely conservative interactions with the sample [29]. The result is that the drive signal image acquired with FM-AFM can show contrast from purely conservative interactions. This is precisely the result found in both the experiments and simulations shown in Fig. 3, where the drive signal shows variations in conservative component of the interaction such as the stiffness of the virus or the conservative van der Waals force.

As shown above FM-AFM has a number of advantages over other more standard AFM modes. We are sure that it will soon become the standard AFM technique used to image weakly attached soft samples immersed in buffer solution with high resolution.

## Materials and Methods

### AFM

The data were taken using a Nanotec Electronics system. The data acquisition and processing was carried out with WSxM [30]. The cantilevers used for the fig. 2c, f and 3a and d are Olympus OMCL-RC800PSA with a nominal force constant of 0.75 N/m. The topographic image of figs. 2e, 3b, c, e and f were acquired with Nanosensors PPP-FMR with a nominal force constant of 2.8 N/m. Figure 2a is taken with a Nanosensors- PPP-NCH cantilever with nominal force constant of 40 N/m. This last type of cantilevers was also used to image viruses in buffer solution (fig. S1. Figs. 4, 5 and 6 were also measured with Olympus OMCL-RC800PSA (we have also obtained true atomic resolution on mica using this type of cantilever see text S1 and fig. S1 and S2).

### Simulations

The simulations were carried out using VEDA [31] [32]. More details on simulation can be found in Text S1 and fig. S3

### Surface preparation

Virus particle adsorption is highly efficient on hydrophobic surfaces. In order to adsorb the virus particles two different hydrophobic surfaces were used: silanized glass surfaces (figs. 2, 3 and 4) and HOPG/figs. 5 and 6). The glass surfaces were by sonicating for 5 minutes in a solution of KOH pellets dissolved in distilled water and ethanol (water∶ethanol ratio was around 1∶9). A special holder was used to properly expose the substrates to the cleaning product. The KOH solution was then removed and the glass surfaces were washed and sonicated again with distilled water for 5 minutes. This process was repeated up to three times. After this, the glass surfaces were dried in a furnace at 100°C. Once the cleaning is done, the substrate holder was left in a closed glass box with a layer of Hexamethyldisilazane [33] that covers the bottom, this ensures that the holder is properly cleaned for further uses. Finally, the glass surfaces were exposed to silane vapor silanes overnight. This process increases the hydrophobicity of the glass increasing the sticking probability of the virus particles to surface. Before the virus particles were adsorbed we checked the surface state by scanning a few substrates with AFM; clean silanized glass surface, suitable for AFM scanning, should be flat, (we measure a roughness smaller than 1 nm) with a few randomly distributed holes of nanometer size.

The HOPG surface where prepared by simply mechanical exfoliation with adhesive tape. This exposed a fresh surface on where a drop of virus solution is deposited.

### Production of virus particles

Purified MVM particles were obtained as described in [10].

φ29 virions were produced as previously described [34], [35] with modifications. Briefly, the *B. subtilis* strain 110NA (*sup* ^−^) was infected with wild-type φ29. The purification of the phage particles was performed using a one-step cesium chloride gradient standard technique (0.75 g/ml cesium chloride in TMS buffer at pH 7.8). The band corresponding to phage particles was extracted and dialyzed against TMS buffer.

## Supporting Information

Text S1 A discussion of technical details of the manuscript can be found in the supplementary information text.
(DOCX)

Figure S1 φ29 topographies obtained with high stiffness cantilevers (nominal stiffness 40 N/m). The dimension of the virus particles can be seen in the lines profiles.
(TIF)

Figure S2 True atomic resolution of a mica surface immersed in a physiological buffer. The cantilever used to acquired the image has a stiffness as low as 0.6 N/m. Oscillation amplitude 0.7 nm, scan speed 600 nm/s, frequency shift 93 Hz.
(TIF)

Figure S3 Feedback scheme for FM-AFM.
(TIF)
