# Supplementary material for: Resolving Structure and Mechanical Properties at the Nanoscale of Viruses with Frequency Modulation Atomic Force Microscopy
Source: PLoS One. 2012 Jan 25;7(1):e30204. doi: 10.1371/journal.pone.0030204 (PMC3266245; doi:10.1371/journal.pone.0030204)
Supplement: Text S1 — A discussion of technical details of the manuscript can be found in the supplementary information text. (DOCX) [file pone.0030204.s001.docx]

**Text S1**

**Supporting information for**

**Resolving structure and mechanical properties at the nanoscale of Viruses with frequency modulation atomic force microscopy**

David Martinez-Martin ^+1^, Carolina Carrasco^+1,2^, Mercedes Hernando-Perez^1^, Pedro J. de Pablo^1^, Julio Gomez-Herrero*^1^, Rebeca Perez^3^, Mauricio G. Mateu ^3^, Jose L. Carrascosa^2^, Daniel Kiracofe ^4^, John Melcher^4^ and Arvind Raman^4^

^1^ Dpto. Física de la Materia Condensada. Universidad Autónoma de Madrid. 28049-Madrid, Spain.

^2^Centro Nacional de Biotecnología. CSIC, 28049 Madrid, Spain.

^3^ Centro de Biología Molecular ‘‘Severo Ochoa’’ (CSIC), 28049-Madrid, Spain;

^4^School of Mechanical Engineering and Birck Nanotechnology Center

Purdue University, West Lafayette, IN 47907

^+^Both authors contributed the same.

*corresponding author: Julio.gomez@uam.es

**Viruses images with high stiffness cantilevers.**

AFM image in liquids have been traditionally performed with low stiffness cantilevers. This is condition is relaxed in FM-AFM where almost any kind of cantilever force constant can be used. Figure S1 shows two good resolved φ29 virus particles taken with a cantilever with a nominal force constant of 40 N/m (Nanosensors Pointprobe NCH). This type of cantilevers is commonly used for AM-AFM in air ambient conditions but it is impossible to obtain reproducible images of weakly attached biological material in liquids for any other AFM mode but FM-AFM. This is indeed the same cantilever used to obtain the atomically resolved images shown in figure 2a. In order to illustrate the advantages of 3D rendering one of the particles is shown in both top view and in a projection view.

**Atomic resolution with low stiffness cantilevers.**

While using low stiffness cantilevers to obtain atomic resolution is not the standard procedure in FM-AFM in liquids we are presenting an atomically resolve mica image (fig. S2) with a soft cantilever (calibrated force constant 0.6 N/m). This type of cantilevers was widely used to obtain virus images in the manuscript.

**Simulation details**

In this section we give more details of the simulator and the mathematical models of cantilever dynamics and tip-sample interaction that it is based on.

The simulations have been performed using a new version of the Virtual Environment for Dynamic AFM (VEDA) simulator [[1](#_ENREF_1),[2](#_ENREF_2)] that incorporates the most relevant features of FM-AFM. VEDA is a freely accessible web-based simulator available on nanoHUB (www.nanohub.org), which is a website dedicated to nanotechnology research and education. nanoHUB offers free online presentations, learning modules, and simulations. A block diagram of the simulator is shown in Figure S3. We shall overview the cantilever dynamics, tip-sample interaction models, and feedback control scheme that are included in the simulations.

The majority of existing FM simulators use a single eigenmode model. This is insufficient to capture the dynamics of soft or moderately stiff soft cantilevers in liquids. Thus, the cantilever dynamics are modeled by a two degree of freedom model [[3](#_ENREF_3)].

where $q_{i}(t),\text{ }\omega_{i},\text{ }k_{i},\text{ }Q_{i},$ and $F_{i}$ are the displacement, natural frequency, modal stiffness, quality factor, and driving force of the *i*th (i=1,2) eigenmode,$d(t)=Z(t)+y(t)+q_{1}(t)+q_{2}(t)$ is the instantaneous tip sample gap, , y(t) is the motion of the dither piezo, and is the tip-sample interaction function.

The tip-sample interaction can be separated into conservative and non-conservative parts.

The conservative model used Figure 4 is a piecewise linear contact model, with one stiffness for representing the solvation shells and a different stiffness for the sample. The force is given by:

$F_{cons}(d)=\left\{\begin{matrix} 0, & d>d_{0} \\ -k_{ts,1}(d-d_{0}), & d_{0}\geq d\geq0 \\ -k_{ts,2}d, & d<0 \end{matrix}\right.$,

where $k_{ts,1}$ and $k_{ts,2}$ are the sample stiffnesses and $d_{0}$ is the distance over which $k_{ts,1}$ acts.

The conservative model used in Figures 5 and 6 is DMT contact [[4](#_ENREF_4)] . In general DMT is valid for relatively stiff contacts with low adhesion, and in liquids it can be used for high buffer concentration solutions where the electrical double layer forces are effectively screened. The force is given by

$$F_{cons}(d)=\left\{\begin{matrix} \frac{-HR}{6d^{2}}, & d>a_{0} \\ \frac{-HR}{6a_{0}^{2}}+\frac{4}{3}E^{*}\sqrt{R}(a_{0}-d)^{3/2}, & d\leq a_{0} \end{matrix}\right.$$

where H, R, and $a_{0}$ are the Hamaker constant, tip radius, and intermolecular distance, respectively, and $E^{*}=\left[\frac{1-\nu_{tip}^{2}}{E_{tip}}+\frac{1-\nu_{sample}^{2}}{E_{sample}}\right]^{-1}$is the effective Young's modulus of the tip-sample combination which can be written in terms of the Young's moduli and Poisson's ratio of the tip and the sample respectively, *E_tip_, ν_tip_, E_sample_, ν_sample_* and *R* is the tip radius.

The simulations in Figures 5 and 6 do not include any non-conservative force. The simulations for these figures use a tip sample dissipation model based on the Kelvin-Voigt viscoelasticity model with a viscous dashpot in parallel to the sample elastic stiffness.

,

where $\eta$ is the viscosity. This is the simplest possible model of viscoelasticity. Comparisons to more sophisticated models [[1](#_ENREF_1)] (not shown) confirm that this model captures the essential features relevant to the discussions in the text.

The feedback control scheme is that of a generic frequency modulated AFM (refer to Figure S3). There is a phase locked loop (PLL) with a PI controller that keeps the cantilever oscillation phase constant and an amplitude feedback loop with a PI controller that keeps the cantilever oscillation amplitude constant. Because only approach curves have been simulated in this paper, there is no Z feedback loop. Additionally, we have explicitly included the effect of piezo-resonances in the excitation scheme. The natural frequency and quality factor of the piezo resonance is deduced by comparing the driven tuning curve with a thermal noise tuning curve. Then the frequency response of the base motion was modeled as a simple harmonic oscillator, and the transfer function between base and tip was as given in [[5](#_ENREF_5)]. This was necessary for quantitative agreement with experiments, but it does not alter the qualitative results (I.e. the conclusions in the main text are still valid for magnetic or photothermal excitation schemes).Aside from the piezo, no attempt has been made to make the controller match specific hardware or the AFM used in the present experiments.

Therefore, the units of the cantilever driving signal channel do not correspond to the units observed in the experiments (volts). The cantilever driving signal channel has been scaled to be unity when the cantilever is not in contact with the sample (i.e. Z > 0). The feedback controller gains and filter time constants have been adjusted to provide reasonable responses but were not tuned to match specific hardware.

**References.**

1. Melcher J, Kiracofe D, Hu S and Raman A (2010). <http://nanohub.org/resources/adac>

2. Melcher J, Hu SQ, Raman A. (2008) Invited Article: VEDA: A web-based virtual environment for dynamic atomic force microscopy. Rev Sci Instrum 79: -.

3. Basak S, Raman A (2007) Dynamics of tapping mode atomic force microscopy in liquids: Theory and experiments. Appl Phys Lett 91.

4. Derjaguin BV, Muller VM and Toporov YP (1975) Effect of Contact Deformations on Adhesion of Particles. Journal of Colloid and Interface Science 53: 314-326.

5. Kiracofe D, Melcher J, and Raman A (2010) Gaining insight into the physics of dynamic Atomic Force Microscopy in complex environments using the VEDA simulator. In preparation.

6. Xu X, Raman A (2007) Comparative dynamics of magnetically, acoustically, and Brownian motion driven microcantilevers in liquids. J Appl Phys 102.
