# Supplementary figures and images for: Resolving Structure and Mechanical Properties at the Nanoscale of Viruses with Frequency Modulation Atomic Force Microscopy
Source: PLoS One. 2012 Jan 25;7(1):e30204. doi: 10.1371/journal.pone.0030204 (PMC3266245; doi:10.1371/journal.pone.0030204)

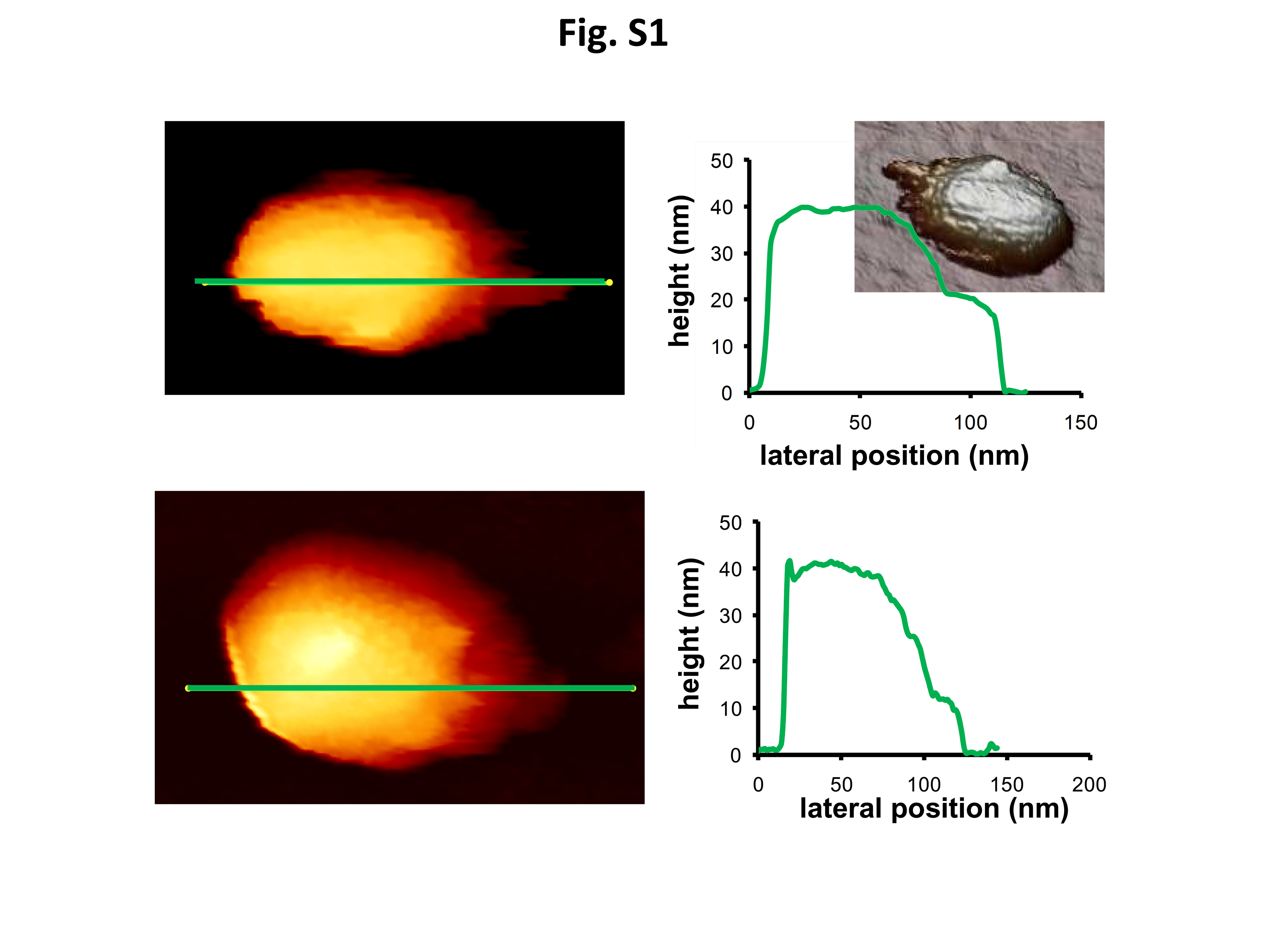

Supplement: Figure S1 — φ29 topographies obtained with high stiffness cantilevers (nominal stiffness 40 N/m). The dimension of the virus particles can be seen in the lines profiles. (TIF) [file pone.0030204.s002.tif]

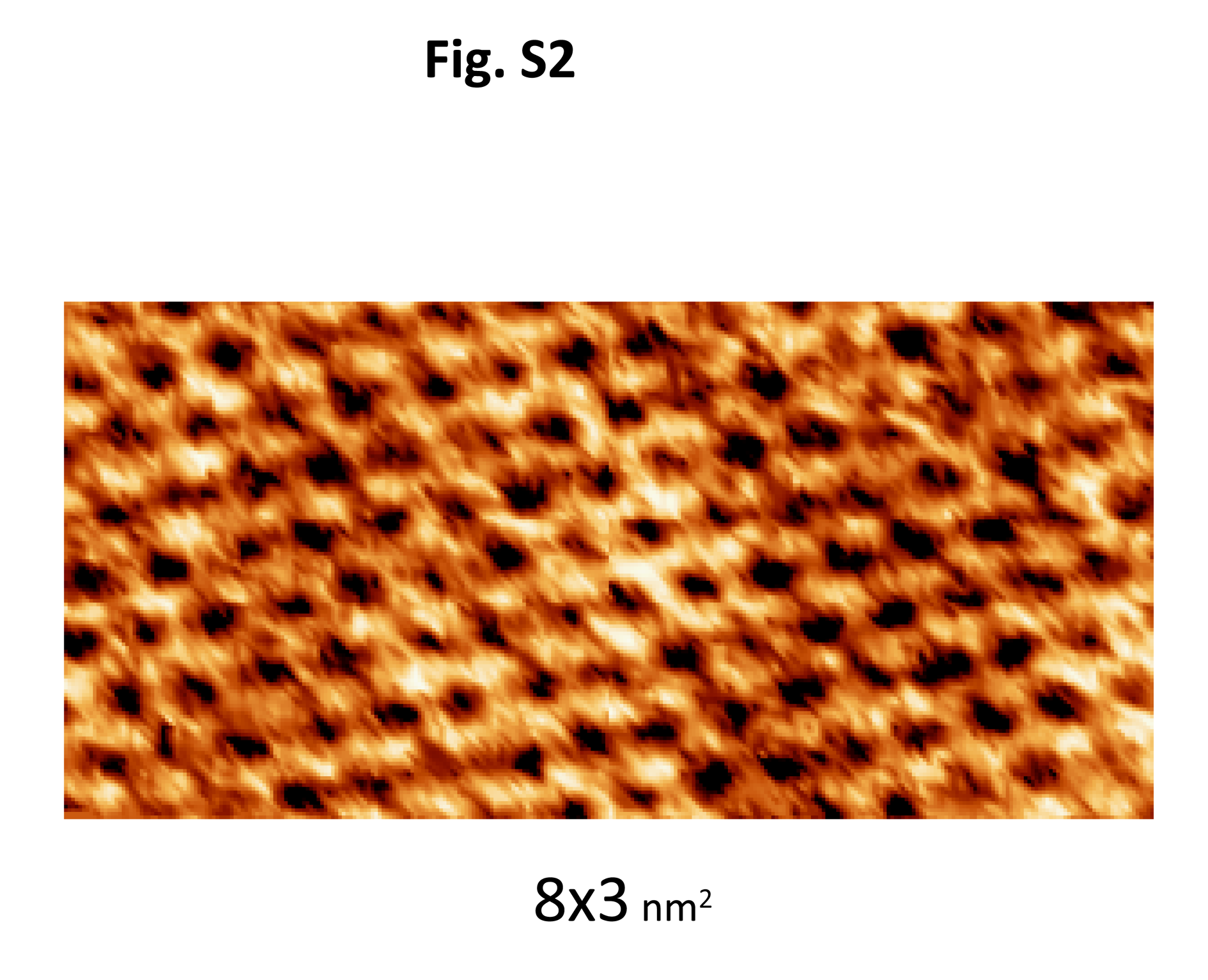

Supplement: Figure S2 — True atomic resolution of a mica surface immersed in a physiological buffer. The cantilever used to acquired the image has a stiffness as low as 0.6 N/m. Oscillation amplitude 0.7 nm, scan speed 600 nm/s, frequency shift 93 Hz. (TIF) [file pone.0030204.s003.tif]

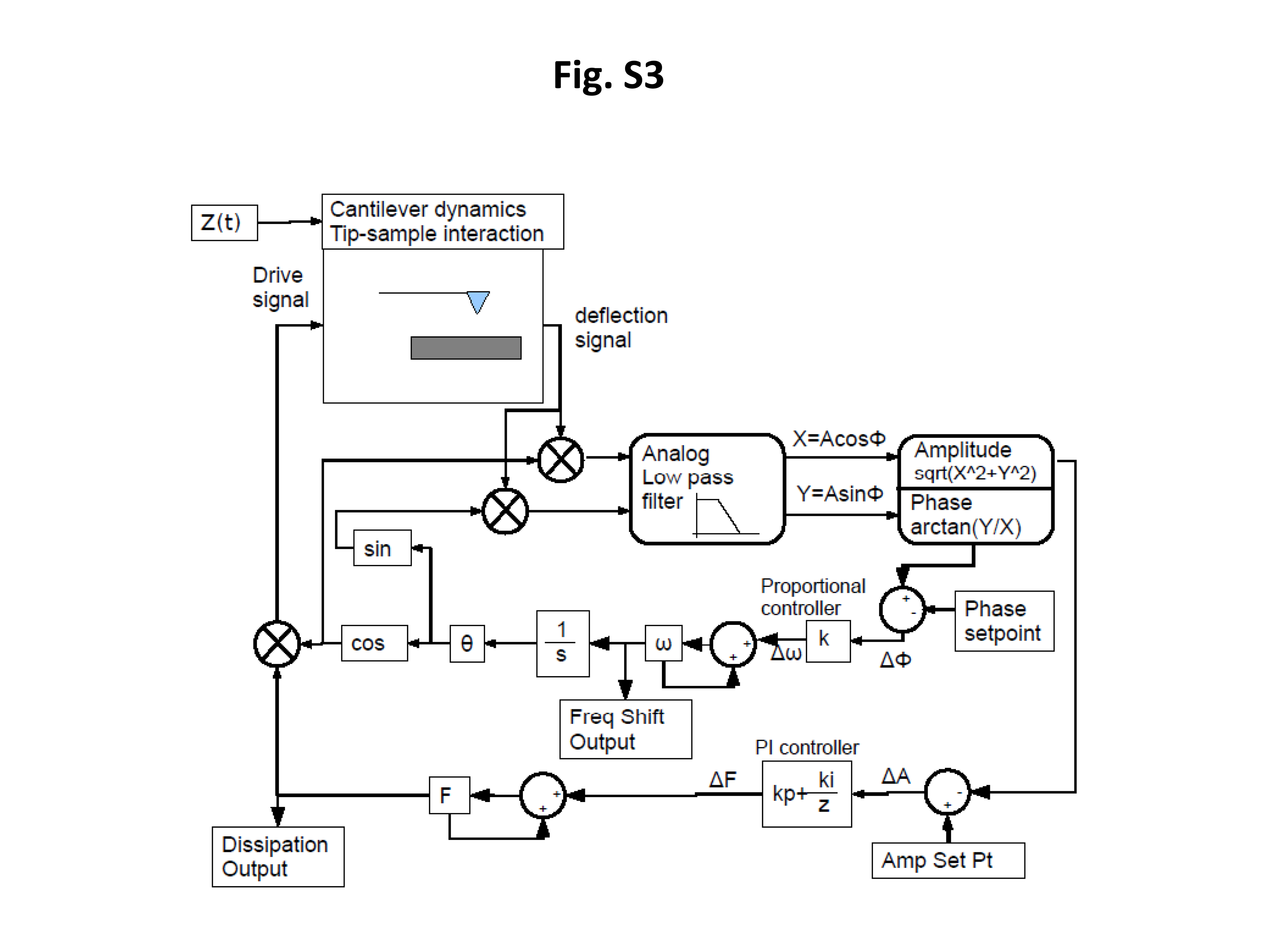

Supplement: Figure S3 — Feedback scheme for FM-AFM. (TIF) [file pone.0030204.s004.tif]
